# Timing social distancing to avert unmanageable COVID-19 hospital surges

**DOI:** 10.1073/pnas.2009033117

**Published:** 2020-07-29

**Authors:** Daniel Duque, David P. Morton, Bismark Singh, Zhanwei Du, Remy Pasco, Lauren Ancel Meyers

**Affiliations:** ^a^Industrial Engineering and Management Sciences, Northwestern University, Evanston, IL 60208;; ^b^Discrete Mathematics, Friedrich-Alexander-Universität Erlangen-Nürnberg, Erlangen 91058, Germany;; ^c^Department of Integrative Biology, The University of Texas at Austin, Austin, TX 78712;; ^d^Operations Research and Industrial Engineering, The University of Texas at Austin, Austin, TX 78712;; ^e^Santa Fe Institute, Santa Fe, NM 87501

**Keywords:** COVID-19, optimization, cocooning, social distancing, public health response

## Abstract

Following the April 16, 2020 release of the *Opening Up America Again* guidelines for relaxing coronavirus disease 2019 (COVID-19) social distancing policies, local leaders are concerned about future pandemic waves and lack robust strategies for tracking and suppressing transmission. Here, we present a strategy for triggering short-term shelter-in-place orders when hospital admissions surpass a threshold. We use stochastic optimization to derive triggers that ensure hospital surges will not exceed local capacity and lockdowns are as short as possible. For example, Austin, Texas—the fastest-growing large city in the United States—has adopted a COVID-19 response strategy based on this method. Assuming that the relaxation of social distancing increases the risk of infection sixfold, the optimal strategy will trigger a total of 135 d (90% prediction interval: 126 d to 141 d) of sheltering, allow schools to open in the fall, and result in an expected 2,929 deaths (90% prediction interval: 2,837 to 3,026) by September 2021, which is 29% of the annual mortality rate. In the months ahead, policy makers are likely to face difficult choices, and the extent of public restraint and cocooning of vulnerable populations may save or cost thousands of lives.

## Significance

How can we best mitigate future pandemic waves while limiting collateral economic damage? As COVID-19 social distancing measures are relaxed across the United States, temporary shelter-in-place orders triggered by monitoring local hospital admissions can minimize the number of days of disruption while preventing overwhelming healthcare surges. We develop a mathematical optimization model on top of an SEIR-style simulation model with age group, risk group, and temporal fidelity. This work has been in response to independent requests from the city of Austin, the state of Texas, the Centers for Disease Control and Prevention, and the White House Coronavirus Task Force to inform strategies for modulating social distancing policies.

As of June 19, 2020, the coronavirus disease 2019 (COVID-19) pandemic continues to spread worldwide and has claimed at least 450,000 lives (1). To avert unmanageable surges in COVID-19 hospitalizations, state and local policy makers across the United States have implemented shelter-in-place orders to enforce social distancing. Under mounting pressures to relieve the economic and societal stresses of shelter-in-place orders, the US White House and Centers for Disease Control and Prevention (CDC) issued *Opening Up America Again* on April 16, 2020, which is a three-phased plan for relaxing such restrictions around the country (2).

In the absence of prophylactic and therapeutic countermeasures, nonpharmaceutical interventions are the only option for mitigating pandemic morbidity and mortality. Measures such as closures of schools and nonessential businesses, prohibitions on public gatherings, requiring social distancing, and restricting travel, along with ordering face covering, frequent hand washing, surface cleaning, and staying at home when sick, can reduce both the frequency and risks of contacts between susceptible and infected people. During the early months of the 1918 influenza pandemic—the only comparably severe pandemic in recent history—aggressive social distancing proved critical to reducing mortality in the United States (3). Despite the life-saving potential of social distancing measures (4, 5), they are controversial (6), given their potentially large economic (7), societal, and mental health (8) costs. Two recent studies have projected pandemic resurgence if social distancing measures are relaxed prematurely (9, 10), and others recommend the gradual relaxation of social distancing measures only when hospitals are no longer overburdened, to balance expected public health risks and economic strain (11–13).

To this end, the goals of this article are threefold. First, we present a conceptual and quantitative framework that clarifies COVID-19 policy options for mitigating risk in the wake of the first pandemic wave. Second, we apply the framework to derive optimal triggers for initiating and relaxing shelter-in-place orders to minimize the number of days of costly social distancing while ensuring that COVID-19 hospitalizations do not exceed local capacity. Finally, we demonstrate the incontrovertible importance of sheltering vulnerable populations to reduce the burden of COVID-19. The impact of future social distancing policies will depend on public adherence, which is highly unpredictable. There are roughly two possible futures. In the first future, the pandemic is held at bay through a combination of public willingness to sustain extreme social distancing despite its costs and a ramping up of testing, contact tracing, and isolation to rapidly contain emerging clusters. In the other future, a relaxation in social distancing or insufficient containment resources allow a second pandemic wave to emerge. For a policy maker facing the latter scenario, either intentionally or unintentionally, our framework provides guidance for enacting short-term lockdowns based on trends in local hospitalization data, to avert unmanageable hospital surges while minimizing social and economic disruption.

Our optimization model, detailed in *SI Appendix*, is designed to guide the relaxation of social distancing. To demonstrate, we derive optimal surveillance triggers for enacting and lifting temporary shelter-in-place orders in the Austin–Round Rock metropolitan area of Texas (henceforth Austin), with a high-risk population of 547,474 and total population of 2,168,316. Austin leadership enacted the *Stay Home – Work Safe Order* (SHWSO) (14) on March 24, 2020, requiring individuals to stay at home except for certain essential activities. The decision to do so was partially based on model projections provided by the University of Texas at Austin. Three weeks later, a second order was issued, requiring cloth face coverings in public spaces. On May 1, 2020, Austin was forced to relax several of these requirements and allow its citizens to return to work, entertainment, and commerce as Texas mandated the first phase of *Opening Up America Again* (15).

The analyses presented herein were requested by the Austin mayor and county judge and are informing ongoing risk assessments, policy planning, and public messaging. We estimate that the COVID-19 epidemic began with a single importation case to Austin on February 15, 2020 and that the March 24 SHWSO reduced transmission by 95% through May 1, as fit using local hospitalization data; see *SI Appendix*. We project pandemic hospitalizations under various intervention scenarios through September 2021. Schools are assumed to remain closed from March 14 until August 18, 2020. After that, they can be reopened or closed in tandem with shelter-in-place orders (16). After May 1, we assume that the city is either in a relaxed state in which the transmission rate is partially but not fully reduced by limited measures and efforts to test, trace, and isolate cases, or a lockdown state in which renewed shelter-in-place orders reduce transmission by 90% relative to the baseline. We find simple triggers for issuing sheltering orders and estimate the impact of cocooning vulnerable populations, that is, maintaining a 95% reduction in transmission to high-risk individuals. These findings provide actionable insights for other metropolitan areas where shelter-in-place orders have curbed the first wave of the COVID-19 pandemic. Moreover, the framework can incorporate any dynamic model of COVID-19 transmission to support similar planning throughout the United States.

## Results

All of our results are based on simulating variable levels of social distancing using a data-driven model for COVID-19 transmission and healthcare needs in the Austin, TX, metropolitan statistical area (MSA) (16). Based on COVID-19 hospitalization data from the Austin–Round Rock MSA through April 16, we estimate that local COVID-19 transmission rates dropped by approximately 95% following the March 24 SHWSO, as detailed in *SI Appendix*. Our simulations assume that Austin maintained this reduced transmission rate until the Texas governor issued the first statewide relaxation order, effective May 1 (17). Following that order, our model allows Austin to toggle between a relaxed state, in which transmission is reduced by 40% relative to the baseline transmission rate estimated prior to schools closing on March 14, and a lockdown state, in which transmission is reduced by an estimated 90%. The relaxed state (40%) does not fully rebound to baseline transmission, under the assumption that testing-based containment and voluntary social distancing will partially mitigate risks. The lockdown state does not quite reduce transmission as much as the original stay-home order—by 90% rather than 95%—to account for likely declines in adherence. Further analyses for other degrees of relaxation, ranging from a 20% to 80% reduction in transmission, are provided in *SI Appendix*. All projections end in September 2021, which is an optimistic time horizon for the development and distribution of prophylactic or therapeutic medical countermeasures (18).

**Fig. 1.**
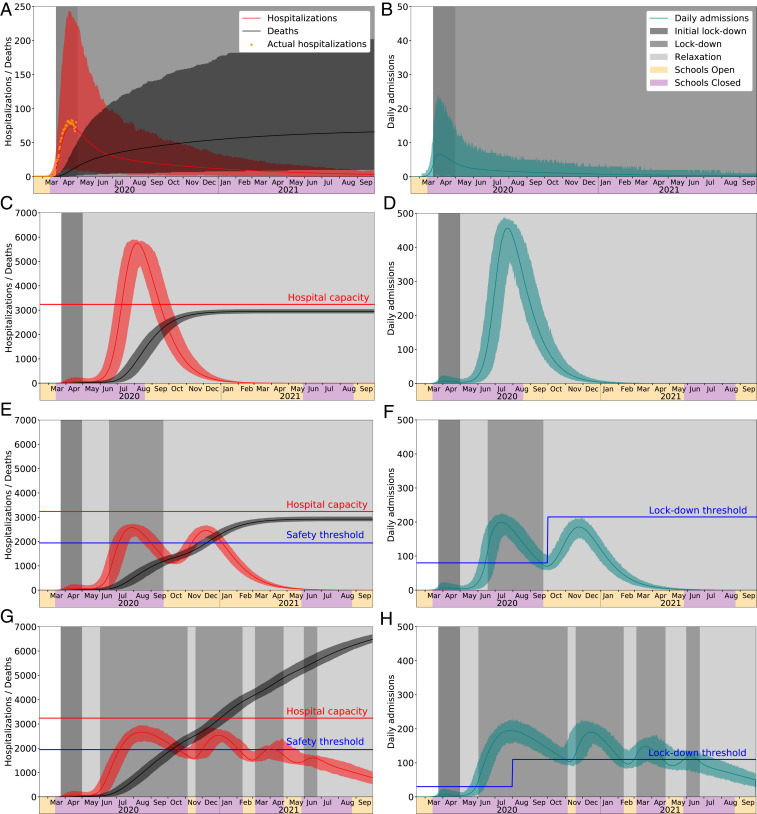
Projections for COVID-19 hospitalizations and deaths in Austin, TX, metropolitan area under baseline and optimized policies for initiating and relaxing social distancing measures. *A*, *C*, *E*, and *G* show daily hospitalizations and cumulative deaths. COVID-19 surge capacity in Austin is approximately 3,240 beds (red line). Daily COVID-19 hospitalizations for the entire metropolitan area from March 13 to April 28 are shown in *A*; data up to April 16 were used to fit the seed date and transmission rates in the model. Optimized strategies can relax lockdowns when total hospitalizations drop below a safety threshold of 60% capacity (blue line). *B*, *D*, *F*, and *H* show daily hospital admissions. Optimized strategies use a stepped threshold: Lockdowns are enacted when the 7-d rolling average in daily admissions surpasses a threshold and are relaxed when admissions decline below a threshold (indicated with blue horizontal lines), if hospitalizations are below 60% capacity. Note that the cyan curves indicate daily admissions rather than 7-d averages, and thus changes (indicated by horizontal gray regions) are triggered a few days after the daily values cross a threshold. (*A* and *B*) The lockdown continues through September 2021, resulting in a 90% reduction in transmission, along with vigilant cocooning of vulnerable populations (95% effective), and school closures. (*C* and *D*) The lockdown is relaxed on May 1, 2020. Thereafter, transmission is reduced by 40%, schools open in mid-August 2020, and 95% effective cocooning of vulnerable populations is maintained through September 2021. Hospitalizations are expected to grossly overrun capacity. (*E* and *F*) Adaptive lockdowns are triggered when hospital admissions cross optimized thresholds, assuming 95% effective cocooning of vulnerable populations. The thresholds minimize the expected days of lockdown while ensuring hospital capacity is not exceeded with high probability. (*G* and *H*) Adaptive lockdowns are triggered when effectiveness of cocooning drops to 80%. Even under an optimized solution, expected deaths and days in lockdown both more than double, relative to cocooning at 95%. In all graphs, solid curves correspond to the point forecast, and shaded regions give 90% prediction intervals based on 300 stochastic simulations.

To evaluate and optimize intervention policies, we compare two outcome measures. First, we measure the total number of days of lockdown (i.e., shelter-in-place) until September 30, 2021 as a proxy for the economic and societal costs of the policy, depicted by gray shading in Fig. 1. Second, we determine the probability of exceeding hospital capacity as a proxy for the public health risks of the policy, indicated when the red hospitalization curves surpass the red capacity line in Fig. 1.

We first project COVID-19 hospitalizations in the extreme scenario that the city maintains a 90% reduction in local transmission indefinitely through a combination of extensive social distancing, transmission-reducing precautions, and proactive testing, contact tracing, and isolation (Fig. 1 *A* and *B*). The analysis assumes that schools remain closed, and cocooning of high-risk populations reduces their risk of infection by 95% rather than 90%. Under this policy, we would not expect a second wave to emerge during the model horizon (Fig. 1*A*). Cumulative deaths would be expected to slowly climb to 81 (90% prediction interval: 10 to 202). This scenario costs a year and a half (555 d) of lockdown.

In the other extreme, consider the scenario in which Austin permanently relaxes social distancing on May 1, while continuing to cocoon high-risk populations and opening schools on August 18 (Fig. 1 *C* and *D*). While this policy requires a lockdown for only the initial 38-d period prior to May 1, we would expect a catastrophic surge in hospitalizations that exceeds the local capacity by 80% during July–September 2020, resulting in an expected 23,075 (90% prediction interval: 22,409 to 23,741) patients not receiving critical care. Without accounting for the excess mortality during this period, which could be considerable, we would expect at least 30-fold higher COVID-19 mortality relative to the indefinite lockdown scenario, with deaths reaching 2,957 (90% prediction interval: 2,868 to 3,040) by September 2021. Under this policy, we expect two epidemic waves during the model horizon, with the second large wave peaking in the late summer of 2020.

Assuming that the first scenario is unattainable and the second scenario unacceptable, we seek alternative policies that limit the number of days in lockdown while preventing COVID-19 healthcare surges beyond local capacity. Based on our decision support efforts for the city of Austin and potential biases in confirmed case count data across the United States, we conjecture that local hospitalization data will be a more reliable indicator of transmission intensity and future hospital surges. Our best policies track daily COVID-19 hospital admissions and daily total hospitalizations across the city and trigger the initiation and relaxation of lockdown periods when admissions cross predetermined thresholds.

Specifically, we formulate and solve a stochastic optimization problem that selects daily hospitalization triggers and recommends reinstatement and relaxation of lockdown periods as follows: 1) reinstate the lockdown—corresponding to a 90% reduction in transmission—when the 7-d average of daily hospital admissions exceeds the trigger; and 2) release the lockdown—corresponding to a 40% reduction in transmission—when both a) the 7-d average of daily hospital admissions drops below the trigger, and b) city-wide hospitalizations (heads in beds) are below a fixed factor (60%) of surge capacity for COVID-19.

We include criterion 2b to hedge against premature relaxation when hospitals are relatively full. If randomized testing becomes available at sufficient scale, we could similarly determine triggers based on testing rather than hospitalization data, and thereby gain earlier indications of a rising or declining threat.

Given the hospital capacity in the Austin, TX, metropolitan area, we recommended a simple, yet robust, strategy with two fixed thresholds, as indicated by the blue step function in Fig. 1*F*. The policy tracks the 7-d moving average of daily COVID-19 hospital admissions and triggers the tightening and loosening of measures when the value crosses 80 daily admissions prior to September 30, 2020, and 215 thereafter. We optimized these two values as well as the date of the transition. Under the point forecast for the pandemic, 135 d of lockdown are required, and hospitalizations remain safely below capacity. Stochastic simulation yields a mean of 135 d (90% prediction interval: 126 to 141). The projected mortality is substantial, with a mean of 2,929 deaths (90% prediction interval: 2,837 to 3,026), which is again over 30 times larger than the baseline scenario of indefinite lockdown (Fig. 1*A*). While the other baseline scenario of indefinite relaxation projected similar COVID-19 mortality (Fig. 1*C*), it produces a catastrophic surge in hospitalizations, and those projections do not account for excess mortality caused by inadequate healthcare resources during the July–September 2020 surge period.

These projections assume an ambitious policy of cocooning vulnerable populations with a 95% level of effectiveness. If cocooning only attains an 80% reduction in transmission risk, then we would expect far greater numbers of hospitalizations and deaths. Under this scenario, the optimal policy requires lower thresholds for enacting lockdowns: a 7-d moving average exceeding 30 daily COVID-19 hospital admissions prior to July 31, 2020 and 110 thereafter (Fig. 1 *G* and *H* and Tables 1–3). Leaky cocooning can substantially undermine containment. In this case, the optimal strategy for managing hospital surge requires multiple periods of lockdown totaling about 350 d and more than doubling expected mortality.

**Table 1.** Projected days of lockdown and COVID-19 mortality under the optimized strategies with 95% and 80% effective cocooning of vulnerable populations

Scenario	Cocooning at 95%	Cocooning at 80%
Days of lockdown		
Mean	135	346
Median	135	347
5 to 95% PI	(126 to 141)	(333 to 360)
Cumulative deaths by		
September 30, 2021		
Mean	2,929	6,527
Median	2,646	6,532
5 to 95% PI	(2,837 to 3,026)	(6,338 to 6,690)


The second and third columns correspond to Fig. 1 *E* and *F* and Fig. 1 *G* and *H*, respectively. Each 90% prediction interval (PI) (i.e., from 5 to 95%) shown in the table and in Fig. 1 is based on 300 simulations.

**Table 2.** COVID-19 mortality under the optimized strategies with 95% and 80% effective cocooning of vulnerable populations

		Percent deaths	
Risk group	Age group	Cocooning at 95%	Cocooning at 80%
Low risk			
	0 y to 4 y	0.03	0.02
	5 y to 17 y	0.20	0.08
	18 y to 49 y	9.01	3.60
	50 y to 64 y	18.23	6.97
	65 y+	4.43	6.12
High risk			
	0 y to 4 y	0.00	0.02
	5 y to 17 y	0.10	0.09
	18 y to 49 y	6.04	5.82
	50 y to 64 y	26.86	28.32
	65 y+	35.06	48.95


The third and fourth columns correspond to Fig. 1 *E* and F and Fig. 1 *G* and *H*, respectively.

**Table 3.** Probabilities of exceeding hospital capacity under the optimized strategies with 95% and 80% effective cocooning of vulnerable populations

	Probability of exceeding percent of hospital capacity	
Hospital capacity	Cocooning at 95%	Cocooning at 80%
60%	1.000	1.000
70%	0.997	1.000
80%	0.803	0.973
90%	0.020	0.197
100%	0.000	0.007


The second and third columns correspond to Fig. 1 *E* and *F* and Fig. 1 *G* and *H*, respectively. Note that Fig. 1 shows 90% prediction intervals (PIs), 5%-95%, for hospitalizations based on 300 simulations, and the last row of the table includes more extreme events.

We conducted sensitivity analyses to assess the robustness and limitations of the optimized triggers. The proposed triggers are relatively robust to weaker social distancing during relaxation periods, for example, if transmission is only reduced by 20% rather than 40%. However, the proposed triggers are not robust to leaky cocooning. We analyze the relative merits of policies with a constant lockdown threshold to the horizon, relative to having two distinct thresholds, as presented here. We show the importance of optimizing trigger thresholds: Conservative triggers significantly increase the duration of lockdown periods, while loose triggers result in hospital capacity being overrun. See *SI Appendix* for details.

## Discussion

A significant relaxation of social distancing in the absence of a comprehensive program for testing, contact tracing, and isolation will likely lead to future waves of the COVID-19 pandemic in US cities. Even if policy makers extend lockdown periods, lack of public willingness to comply might undermine their efficacy. Thus, planning for future relaxations is paramount to averting unmanageable surges in COVID-19 hospitalizations. Carefully designed strategies for triggering future shelter-in-place measures can mitigate the impact on the community’s healthcare system while minimizing economic and societal costs.

Our framework clarifies a key decision facing city and state leaders in the wake of the first wave of COVID-19—when to enact and relax social distancing measures should the epidemic rebound. We posit a simple strategy for measuring and responding to future surges in hospitalizations—enact and then relax temporary lockdowns when daily hospital admissions climb above and eventually recede below a predetermined, optimized threshold. Whereas policy makers and public dashboards primarily track confirmed COVID-19 cases and deaths, we intentionally use COVID-19 hospitalization data to fit our model parameters and guide policy. Hospitalization admissions are less subject to bias than confirmed case counts, given the heterogeneous and rapidly changing test availability and priorities across the United States, and they are less time-lagged than reported COVID-19 deaths. Our policies are specifically designed to track a 7-d rolling average for daily COVID-19 hospital admissions, to prevent overreacting to statistical variability and to account for weekly periodicity in hospital reporting.

The optimal strategies derived for Austin, TX, provide three critical insights. First, data-driven optimization yields policies that are expected to protect against catastrophic hospital surges while requiring far fewer days of costly shelter-in-place measures than most sensible expert-designed strategies. For example, triggering lockdowns based on an arbitrarily chosen trigger of 50 new admissions per day should prevent hospitalizations from reaching capacity, but they are expected to require more than 150 additional days of lockdown, relative to the optimized trigger policy. However, implementing this trigger-based optimization framework requires continual review of daily hospital admissions and overall hospital utilization, as well as constant validation of transmission rates during lockdown and relaxation phases.

Second, under the plausible scenario that transmission rebounds to 60% of baseline (i.e., a 40% reduction), the best strategy for limiting lockdowns without undermining the healthcare system would likely trigger only one future lockdown in mid-June following a steep increase in hospitalizations that surpasses the trigger of 80 new admissions per day (Fig. 1*F*). Hospitalizations would then be expected to peak and subside in late July, allowing relaxation of the lockdown by late September. The simultaneous release of the lockdown and start of a delayed 2020–2021 school year would fuel a third wave, which would be expected to be self-limiting, that is, subside without requiring a third lockdown period. This decline is driven by herd immunity, with an expected 79% of the population already infected and recovered by October 2021.

We emphasize that, while this strategy offers a practical balance between economic and healthcare constraints, it is not designed to minimize morbidity and mortality and results in nearly 3,000 expected deaths by September 2021. If we assume a similar COVID-19 mortality rate for the entire United States, this extrapolates to over 450,000 deaths, an order of magnitude higher than the annual mortality from seasonal influenza. We note, with concern, that this alarming projection assumes that high-risk populations maintain 95% effective social distancing through September 2021.

Finally, failing to vigilantly cocoon our vulnerable populations will significantly increase both the death toll and the requisite number of days in lockdown, even under the most efficient policy for keeping hospitalizations in check. Nursing homes and populations experiencing homelessness have both large proportions of high-risk individuals and living conditions that exacerbate the risks of COVID-19 transmission. Proactive measures to prevent COVID-19 introductions into these communities and to rapidly contain initial clusters are essential to effective cocooning but will require considerable forethought and resources, including additional trained staff and isolation facilities. In addition, providing incentives and support for high-risk members of the workforce to shelter at home will be critical.

While we believe that our qualitative findings are robust and provide actionable insights for navigating the challenges ahead, our quantitative findings are specific to Austin and are based on several simplifying assumptions. For example, we do not consider the impact of the 2020–2021 influenza season on surge capacity for COVID-19 cases. During the 2019–2020 influenza season, several Austin area hospitals neared their capacity. In reality, we cannot predict when or how much transmission will rebound from policy loosening or public fatigue. Yet, our optimal policies assume a specific and constant degree of relaxation. Our sensitivity analysis suggests that the derived policies are relatively robust to uncertainty regarding future transmission but not to a relaxation of cocooning. While our epidemiological model considers age-specific contact rates and vulnerabilities to COVID-19, it does not explicitly model subgroups with anomalously high contact rates, such as nursing home residents, individuals experiencing homelessness, and the healthcare, grocery, and construction workforces. Such high-risk communities can amplify transmission and lead to rapid spikes in hospitalizations that are unmanageable, even when total hospitalizations are far below capacity, or can inadvertently trigger unnecessary lockdowns. We assume a single infectious individual seeded the epidemic in Austin. This does not preclude earlier importations that produce a limited cluster of cases. Fig. 1*A* shows fit and forecast hospitalizations from March 13 through April 16, and additional hospitalizations through April 28. As a final caveat, we note that locking down and opening up a city may require overcoming substantial societal inertia. However, our analyses do not consider potential delays in community responses following a policy change. While we could modify the analysis to build in buffers, we also advocate for the design of multistage “track and trigger” policies that require both less extreme transitions and proactive socialization of such policies by city leaders to cultivate a sense of personal responsibility and accelerate adherence when restrictions are enacted.

Our study presents a relatively simple scenario in which a city can toggle between two extreme states of lockdown and relaxation. It is meant to serve as a proof-of-concept and provide a flexible framework for guiding COVID-19 policies as the pandemic unfolds in cities across the United States. For example, we have adapted this method to help the city of Austin design a five-stage risk chart that allows the city to tap on the brakes to avoid full-blown stay-home measures. Working closely with Austin’s Executive COVID-19 Task Force—including the Austin mayor, Austin Health Authority, judge of the largest county, and leadership from all area hospitals—we derived robust triggers to guide the transitions between all five stages. The city leadership has undertaken an aggressive socialization process to explain when and how behavioral changes will be expected from the city’s citizens. This includes almost daily reminders in the press and a public dashboard that tracks hospital admissions relative to color-coded thresholds provided by our analyses (19). We are monitoring the situation on a daily basis. If local COVID-19 transmission deviates from our original assumptions, our analysis pipeline is poised to rapidly reevaluate the policy options. We emphasize that such triggers should guide rather than dictate policy. For example, if a localized outbreak in a nursing home leads to a surge, but the outbreak does not appear to represent larger trends, city leaders could delay enacting a recommended lockdown.

Our simple threshold policies allow optimization using a relatively small discrete grid. This approach can be directly applied to other epidemiological simulation models, provided that they can incorporate both triggers and variable levels of social distancing, for example, by adjusting transmission rates over time. Modelers can readily incorporate critical subpopulations, like schools, the concurrent transmission of influenza, and intercity travel, all of which may grow in importance in the months ahead. Thus, modelers can broadly apply this framework to provide decision support for COVID-19 responses in cities worldwide. In addition to tracking hospitalizations for triggering shelter-in-place orders, modelers will need to regularly estimate local transmission rates as policies and individual behavior evolve. Integrating cell phone mobility data reflecting social distancing, as we do in our forecasting model (20, 21), may improve the accuracy and timeliness of our estimates. Finally, September 2021 is a long horizon. In the months ahead, the likelihood and timeline for promising antiviral drugs and vaccines may become clearer. If such life-saving measures appear within reach, communities may have a renewed willingness to shelter in place that can be directly incorporated into designing new triggers for aggressive mitigation.

## Materials and Methods

We use a data-driven SEIR (susceptible, exposed, infected, recovered)-style metapopulation model for COVID-19 transmission (16). This epidemiological model has compartments for susceptible, exposed, infectious–asymptomatic, infectious–symptomatic, infected–hospitalized, recovered, and deceased. We partition the population into 10 groups comprising all combinations of five age groups and two risk groups. Contact matrices encode the expected number of daily contacts during a lockdown, and in the relaxed state, and further account for cocooning of high-risk groups, weekdays versus weekends, and whether school is currently open and, if so, the school calendar. Using a least-squares method, we fit the epidemiological model to hospitalization data from March 13 to April 16 via the seed date, a baseline transmission rate, and the reduction in the transmission rate from baseline during SHWSO, with the fit shown in Fig. 1*A*.

We formulate an optimization model that determines daily values for both thresholds in a two-tiered policy depicted in Fig. 1 *F* and *H*. The optimization model has constraints that account for the epidemiological dynamics and a set of constraints that link that model’s 7-d moving average of daily hospital admissions with the trigger thresholds, and the corresponding reductions in transmission via the contact matrices. A final set of constraints keep estimated hospitalizations within capacity. To do so, we use the square-root staffing rule from queueing theory (22). This rule maintains a high probability (we use ≥0.9999) that a single arrival in steady state does not have to wait for service, and yet servers are highly utilized. Our “servers” are hospital beds, along with necessary healthcare providers and equipment. We assume that 80% of Austin’s hospital beds are available for COVID-19 patients. We require that the square-root staffing rule hold, under a point forecast for daily COVID-19 hospitalizations. In addition, we simulate, and optimize with respect to, 300 sample paths of the epidemic, taking into account both macrolevel and microlevel stochastics, with details in *SI Appendix*. We ensure that the probability of exceeding hospital capacity within the time horizon is, at most, 0.01. With these constraints in place, we select triggers to minimize the expected number of days of lockdown. Minimizing lockdown acknowledges social and economic pressures to relax stringent measures.

All data required for this analysis can be found in *SI Appendix*. The code that produced the results of our analyses is available at https://github.com/dukduque/COVID-TriggerOpt.

## Supplementary Material

Supplementary File
