# Supplementary material for: Timing social distancing to avert unmanageable COVID-19 hospital surges
Source: Proc Natl Acad Sci U S A. 2020 Jul 29;117(33):19873–8. doi: 10.1073/pnas.2009033117 (PMC7443931; doi:10.1073/pnas.2009033117)
Supplement: Supplementary File [file pnas.2009033117.sapp.pdf]

# Supplementary Information for

## Timing Social Distancing to Avert Unmanageable COVID-19 Hospital Surges

**Daniel Duque, David P. Morton, Bismark Singh, Zhanwei Du, Remy Pasco, Lauren Ancel Meyers**

**David P. Morton.**
**E-mail: david.morton@northwestern.edu**

### This PDF file includes:

Supplementary text
Figs. S1 to S6
Tables S1 to S15
References for SI reference citations



www.pnas.org/cgi/doi/10.1073/pnas.2009033117

## Supporting Information Text

**Material and Methods.** We optimize the timing of when to initiate and relax local lock-downs (i.e., extreme social distancing measures) via triggers that monitor both daily new hospital admissions and total hospitalizations. A lock-down is triggered when a seven-day moving average of daily hospital admissions grows to exceed a *lock-down threshold*, which we specify for each day. Relaxation of a lock-down is triggered when: (a) the same moving average drops below the lock-down threshold and (b) the total hospitalizations are under a *safety threshold*. Here, lock-down corresponds to high social distancing (SD) —90% reduction in transmission in our instances—and a relaxation of SD means a lower level of SD—we use 40% reduction as a nominal value.

In addition to epidemiological constraints governing the transmission and severity of the virus, our optimization model selects triggers that ensure the aggregate daily arrival rate of new patients to hospitals is such that, with high probability, the demand for hospital beds does not exceed supply. We use the square-root staffing rule of (1) for an $M/M/s$ queue with $s$ servers. That rule says that under a service rate of $\mu$, if the arrival rate, $\lambda$, is large then we require $s \approx (\lambda/\mu) + k\sqrt{(\lambda/\mu)}$ servers to maintain a high probability that the servers are highly utilized and yet an arrival does not have to wait for service. The rule is based on an approximation, which holds in steady state under independent and exponentially distributed interarrival times and service times, and the approximation is increasingly accurate as $\lambda$ grows large. The value of parameter $k$ determines the model's probability that an arriving patient does not have to wait for a hospital bed, and we use $k = 4$, which corresponds to a probability of over 0.99997 for a single patient. We first describe a deterministic optimization model: Among all feasible triggers, the optimization model seeks triggers to minimize the number of days in lock-down.

To find the lock-down and safety thresholds, we formulate an optimization model that determines daily values for both thresholds, while hospitalizations and daily hospitalizations are characterized via an enhanced SEIR-style metapopulation model of disease transmission (2). The underlying epidemiological model comprises compartments for susceptible, exposed, infectious-asymptomatic, infectious-symptomatic, infected-hospitalized, recovered, and deceased, which we denote by $S$, $E$, $IA$, $IY$, $IH$, $R$, and $D$, respectively. Figure S1 diagrams the transitions between the compartments in this model. The population is partitioned into 10 groups comprised of all combinations of five age groups and two risk groups. Each group is represented with its own set of compartments (susceptible, exposed, etc.) so that in total the epidemiological model has 70 compartments. To formalize our model, let $X_t$ be a binary indicator variable that takes the value of one if a lock-down is in place on day $t$. The goal is to find thresholds $l_t$ and $r_t$ for all $t \in \mathcal{T}$ so that the sum of indicator variables is minimized while respecting hospital capacity and epidemiological dynamics.

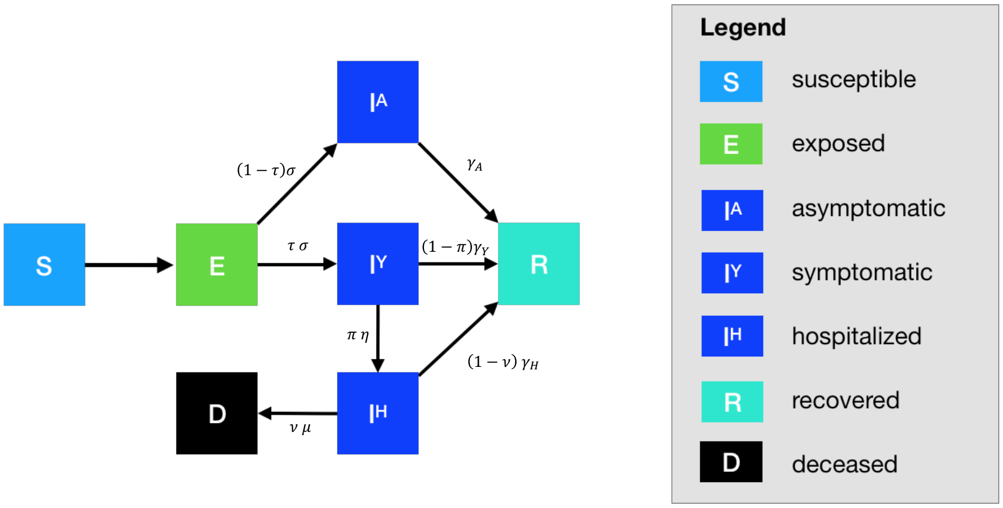


**Fig. S1.** Diagram of the epidemiological variables

<u>Notation:</u>

*Indices and Sets*

$t \in \mathcal{T}$ set of time periods $\{1, 2, \ldots, |\mathcal{T}|\}$ [days]

| | |
|---|---|
| $t \in \mathcal{T}_0$ | $\mathcal{T} \cup \{0\}$ |
| $a \in \mathcal{A}$ | set of age groups {0-4y, 5-17y, 18-49y, 50-64y, 65y+} |
| $r \in \mathcal{R}$ | risk groups {low-risk, high-risk} |
| *Parameters* | |
| *Epidemiological parameters*: | |
| $\beta$ | transmission rate |
| $\sigma$ | rate at which exposed individuals become infectious |
| $\tau$ | proportion of exposed individuals who become symptomatic |
| $\gamma_A$ | recovery rate from asymptomatic compartment |
| $\gamma_Y$ | recovery rate from symptomatic compartment |
| $\gamma_H$ | recovery rate from hospitalized compartment |
| $\eta$ | hospitalization rate after symptom onset |
| $YHR^{a,r}$ | percent of symptomatic infectious that go to the hospital for $a, r$ |
| $\pi^{a,r}$ | $\gamma_Y \cdot YHR^{a,r}/[\gamma_Y \cdot YHR^{a,r} + \eta(1 - YHR^{a,r})]$: rate adjusted proportion for $a, r$ |
| $\mu$ | rate from hospitalization to death; $\mu = \gamma_H$ in our instances |
| $HFR^a$ | percent of hospitalized that die for $a, r$ |
| $\nu^a$ | $\gamma_H \cdot HFR^a/[\gamma_H \cdot HFR^a + \mu(1 - HFR^a)]$: rate adjusted proportion for $a, r$ |
| $\phi_{a',r'}^{a,r,t,high/low}$ | expected number of daily contacts from $(a', r')$ to $(a, r)$ at $t$ under high or low SD |
| $N_{a,r}$ | population of age-risk group $a, r$ |
| $\omega_{A/E}^{a,r}$ | infectiousness of individuals in $IA$ and $E$ relative to $IY$ for $a, r$ |
| *Other parameters*: | |
| $B$ | number of hospital beds for COVID-19 patients |
| $k$ | safety factor for square-root staffing rule |
| $\bar{\lambda}$ | largest $\lambda$ satisfying $\lambda/\mu + k\sqrt{\lambda/\mu} \leq B$ |
| *Variables* | |
| *Epidemiological variables*: | |
| $S_t^{a,r}$ | number of susceptible people of age group $a$, risk group $r$ at time $t$ [persons] |
| $dS_t^{a,r}$ | $S_t^{a,r} - S_{t+1}^{a,r}$ [persons] |
| $E_t^{a,r}$ | number of exposed people of age group $a$, risk group $r$ at time $t$ [persons] |
| $IA_t^{a,r}$ | number of infectious-asymptomatic people for $a, r, t$ [persons] |
| $IY_t^{a,r}$ | number of infectious-symptomatic people for $a, r, t$ [persons] |
| $IH_t^{a,r}$ | number of infected-hospitalized people for $a, r, t$ [persons] |
| $R_t^{a,r}$ | number of recovered people for $a, r, t$ [persons] |
| $D_t^{a,r}$ | number of deceased people for $a, r, t$ [persons] |
| $IYIH_t$ | daily hospitalizations at time $t$ [persons/day] |
| $\overline{IYIH}_t$ | seven-day moving average of $IYIH_t$ [persons/day] |
| *Intervention variables*: | |
| $X_t$ | 1 if lock-down (e.g., 90% SD) is applied at time $t$; 0 otherwise (e.g., 40% SD) |
| $l_t$ | the lock-down trigger for the daily hospitalization rate at $t$ |
| $r_t$ | the safety trigger for total hospitalizations |
| $\phi_{a',r'}^{a,r,t}(X_t)$ | expected number of daily contacts at $t$ determined by $X_t$ |

Epidemiological dynamics:

$$S_{t+1}^{a,r} - S_t^{a,r} = -dS_t^{a,r} \quad \forall t \in \mathcal{T}_0, a \in \mathcal{A}, r \in \mathcal{R} \tag{1a}$$

$$E_{t+1}^{a,r} - E_t^{a,r} = dS_t^{a,r} - \sigma E_t^{a,r} \quad \forall t \in \mathcal{T}_0, a \in \mathcal{A}, r \in \mathcal{R} \tag{1b}$$

$$IA_{t+1}^{a,r} - IA_t^{a,r} = (1-\tau)\sigma E_t^{a,r} - \gamma_A IA_t^{a,r} \quad \forall t \in \mathcal{T}_0, a \in \mathcal{A}, r \in \mathcal{R} \tag{1c}$$

$$IY_{t+1}^{a,r} - IY_t^{a,r} = \tau\sigma E_t^{a,r} - (1-\pi^{a,r})\gamma_Y IY_t^{a,r} - \pi^{a,r}\eta IY_t^{a,r} \quad \forall t \in \mathcal{T}_0, a \in \mathcal{A}, r \in \mathcal{R} \tag{1d}$$

$$IH_{t+1}^{a,r} - IH_t^{a,r} = \pi^{a,r}\eta IY_t^{a,r} - (1-\nu^a)\gamma_H IH_t^{a,r} - \nu^a \mu IH_t^{a,r} \quad \forall t \in \mathcal{T}_0, a \in \mathcal{A}, r \in \mathcal{R} \tag{1e}$$

$$R_{t+1}^{a,r} - R_t^{a,r} = \gamma_A IA_t^{a,r} + (1-\pi^{a,r})\gamma_Y IY_t^{a,r} + (1-\nu^a)\gamma_H IH_t^{a,r} \quad \forall t \in \mathcal{T}_0, a \in \mathcal{A}, r \in \mathcal{R} \tag{1f}$$

$$D_{t+1}^{a,r} - D_t^{a,r} = \nu^a \mu IH_t^{a,r} \quad \forall t \in \mathcal{T}_0, a \in \mathcal{A}, r \in \mathcal{R} \tag{1g}$$

$$dS_t^{a,r} = S_t^{a,r} \sum_{a' \in \mathcal{A}} \sum_{r' \in \mathcal{R}} \frac{\beta \phi_{a',r'}^{a,r,t}(X_t)}{N_{a',r'}} \Big( IY_t^{a',r'} + \omega_A^{a',r'} IA_t^{a',r'} + \omega_E^{a',r'} E_t^{a',r'} \Big) \quad \forall t \in \mathcal{T}, a \in \mathcal{A}, r \in \mathcal{R} \tag{1h}$$

$$\phi_{a',r'}^{a,r,t}(X_t) = \phi_{a',r'}^{a,r,t,high} X_t + \phi_{a',r'}^{a,r,t,low}(1-X_t) \quad \forall a, a' \in \mathcal{A}, r, r' \in \mathcal{R}, t \in \mathcal{T} \tag{1i}$$

Initial conditions:
$IY_0^{18\text{-}49,low} = 1$, $S_0^{18\text{-}49,low} = N_{18\text{-}49,low} - 1$, and $S_0^{a,r} = N_{a,r} \forall a \in \mathcal{A}, r \in \mathcal{R}$.

The initial conditions specify a single infectious individual in the 18-49 age group with low risk. The age-risk groups are initialized with the rest of the population in their respective susceptible compartments. Equations [1a]-[1h] then provide a point forecast for the evolution of the disease. Variable $X_t \in \{0,1\}$ is a toggle that either selects the expected number of daily contacts according to the lock-down, or the relaxed, contact matrix: Parameter $\phi_{a',r'}^{a,r,t,high}$ encodes the expected number of daily contacts on day $t$ when we are in lock-down, i.e., high SD (e.g., 90% SD), and $\phi_{a',r'}^{a,r,t,low}$ does the same for relaxed SD (e.g., 40% SD). These parameters are indexed by $t$ because they capture whether school is currently open and if so, the school calendar; and, they further capture weekdays versus weekends and the level of cocooning, which can vary with time. Hospitalized cases are labeled as "infected hospitalized." We assume that sufficient precautions are taken in hospitals so that hospitalized cases do *not* contribute to infecting others via Eq. [1h].

Threshold policy:

The $X_t$ toggle is determined by the thresholds $l_t$ and $r_t$ using the following logic:

$$IYIH_t = \sum_{a \in \mathcal{A}} \sum_{r \in \mathcal{R}} \pi^{a,r} \eta IY_t^{a,r} \quad \forall t \in \mathcal{T} \tag{2a}$$

$$\overline{IYIH}_t = \frac{1}{7} \sum_{t'=t-6}^{t} IYIH_t \quad \forall t \in \mathcal{T} \tag{2b}$$

$$X_t \geq 1_{\{\overline{IYIH}_t \geq l_t\}} \quad \forall t \in \mathcal{T} \tag{2c}$$

$$X_t \geq 1_{\{X_{t-1}=1 \ \& \ IH_t \geq r_t\}} \quad \forall t \in \mathcal{T} \tag{2d}$$

$$X_t \leq 1_{\{X_{t-1}=1 \ \& \ IH_t \geq r_t\}} + 1_{\{\overline{IYIH}_t \geq l_t\}} \quad \forall t \in \mathcal{T} \tag{2e}$$

$$1 - (X_{t-1} - X_t) \geq \frac{1}{14} \sum_{t'=t}^{t+13} X_{t'} \quad \forall t \in \mathcal{T} : t \geq 2 \tag{2f}$$

$$X_t \in \{0, 1\}, \quad \forall t \in \mathcal{T}. \tag{2g}$$

The systems from Eqs. [1] and [2] are coupled. Given $X_t$ we can specify a solution to Eqs. [1], and that solution defines the arrival rate to the community's hospitals via [2a]. The seven-day moving average of daily arrivals is defined in Eq. [2b]. Given thresholds, Eq. [2c] enforces a lock-down when that average is at or above the lock-down threshold, $l_t$. If the average is below $l_t$ then Eq. [2d] ensures we are still in lock-down if we were yesterday *and* the total number currently hospitalized exceeds the safety threshold. If neither of those events holds then Eq. [2e] requires that we relax SD. To avoid impractical policies that lock-down and relax for short periods of time, Eq. [2f] requires that a relaxation last at least fourteen days if enacted. Given the corresponding $X_t$, Eq. [1i] again selects the corresponding daily-contact matrix, coupling the systems.

Deterministic optimization model:

$$\begin{aligned} \min \quad & \sum_{t \in \mathcal{T}} X_t && \text{[3a]} \\ \text{s.t.} \quad & IYIH_t \leq \bar{\lambda}, \quad \forall t \in \mathcal{T} && \text{[3b]} \\ & \text{Eqs. [1]} && \text{[3c]} \\ & \text{Eqs. [2]} && \text{[3d]} \\ & l_t, r_t \geq 0, \quad \forall t \in \mathcal{T}. && \text{[3e]} \end{aligned}$$

A solution to model [3] provides daily thresholds to guide both lock-down and relaxation of strict SD. Constraint [3b] enforces the square-root staffing rule, and the model's objective is to minimize the number of days in lock-down.

Let $(l, r)$ denote the vector of thresholds. Then we let:

$$\mathcal{F} = \{(l, r) \geq 0 : \text{there exists a solution to Eqs.[1], [2], [3}b\text{]}\}.$$

In other words, $\mathcal{F}$ specifies the set of thresholds that ensure the arrival rate to hospitals is within the rate specified by the square-root staffing rule.

The deterministic finite-difference Eqs. [1], or their differential equation counterparts, are approximations in which binomial random variables replace terms like $\sigma E_t^{a,r}$; here the binomial random variable has parameter $n = E_t^{a,r}$ and $\sigma$ serves as the "success" probability. This construct is pervasive throughout right-hand side terms in Eqs. [1]. In addition to these "micro" stochastics there are "macro" stochastics including modeling $\sigma$ and $\gamma_Y$ and coupled parameters as random variables; see Table S7 for further details. As a result, except for $(l, r)$ we can parameterize all variables by $(S(\omega), E(\omega), IA(\omega), IY(\omega), IH(\omega), R(\omega), D(\omega))$, where $\omega \in \Omega$ indexes a sample path of the stochastic simulation. In this setting we solve the following model.

Stochastic optimization model:

$$\min_{(l,r)} \quad E_\omega \sum_{t \in \mathcal{T}} X_t(\omega) \tag{4a}$$

$$\text{s.t.} \quad (l,r) \in \mathcal{F} \tag{4b}$$

$$\text{Equations [1]} \quad \forall \omega \in \Omega \tag{4c}$$

$$\text{Constraints [2]} \quad \forall \omega \in \Omega \tag{4d}$$

$$P_\omega \left( \max_{t \in \mathcal{T}} \sum_{a \in \mathcal{A}, r \in \mathcal{R}} IH_t^{a,r}(\omega) \leq B \right) \geq 1 - \varepsilon. \tag{4e}$$

Constraint [4b] ensures the thresholds satisfy the square-root staffing rule for the best-guess (deterministic) epidemiological parameters. Constraints [4c]-[4d] track stochastic disease dynamics for each sample path. Constraint [4e] is a probabilistic constraint, which ensures that over the time horizon the maximum number of heads in beds is within hospital capacity for COVID-19 patients with high probability; we use $\varepsilon = 0.01$ in our computation. Subject to these constraints we minimize the expected number of days in lock-down in [4a]. We do not compute exact expectations, but rather solve a sample average approximation of model [4] using Monte Carlo simulation to generate sample paths.

Having a different threshold each day is unrealistic for practical policy implementation. We seek a simpler policy, and so we restrict attention to policies with lock-down thresholds, $l_t$, that can change only *once* during the planning horizon. This class of thresholds can be characterized with three parameters: (i) the threshold value up until the change, (ii) the threshold value after the change, and (iii) the timing of the change. In addition, in our implementation we required a single safety threshold $r = r_t$ for the entire horizon.

The deterministic model of Eqs. [3] can be formulated as a nonlinear mixed-integer program, and model [4] is a stochastic nonlinear mixed-integer program. In its general form, even the deterministic model is challenging to solve with commercial solvers, in part, because of the bilinear terms involving products of continuous variables that appear in Eq. [1h]. (These are further multiplied by binary variables, but those can be linearized exactly.) However, the class of policies just specified simplifies the task of finding an optimal solution, which involves just four decision variables: three associated with $l_t$ and one associated with $r_t$. We optimize over the space of these variables as follows: For the lock-down threshold we parameterize the search on a grid, where the first two decision variables can take values between 0 and $\bar{\lambda}$ in steps of 10 patients per day, and the third decision variable can take values corresponding to the first day of every month. We similarly form a grid for the safety threshold $r$. To select an optimal policy, we first enumerate all possible tuples of the four decision variables and preclude those that do not satisfy the staffing rule defined in Eq. [3b], applying the systems of Eqs. [1]–[2] so that contact matrices are selected on a daily basis according to how the triggers unfold in a deterministic simulation under best-guess parameter values. To obtain an optimal solution to model [4], we run stochastic simulations for each configuration, and then preclude those that do not satisfy inequality [4e], finally selecting the solution with the smallest number of expected days in lock-down.

The trigger-based policy is closed-loop, i.e., adaptive, in the sense that the timing of lock-down and relaxation depends on the sample path of the simulations. All results presented in the main text use 300 stochastic simulations to compute the threshold policy, and an independent set of 300 out-of-sample simulations to report performance of that policy. The initial conditions specify a single infectious individual. As a result, stochastic simulations sometimes produce sample paths that quickly die out. In performing our Monte Carlo sampling, we reject samples that die out since they are inconsistent with observed hospitalizations. Hence, we have to generate more than 300 total sample paths to obtain 300 acceptable sample paths. We use a crude filter that simply requires at least 100 total hospitalizations over the time horizon to September 2021.

We further investigated other classes of thresholds for the lock-down trigger, such as constant, linear, and quadratic thresholds as a function of time. While the step-function threshold for $l_t$ involves three parameters, a constant threshold is parameterized with one value, a linear threshold with two values (intercept and slope), and a quadratic threshold with three parameters (where the third parameter is the coefficient of the quadratic term). Using such restricted classes of thresholds provides policies that are both simple to implement and simple to optimize via simulation for all configurations on a discretized grid. This observation is important because it allows modelers to use our optimization framework with their own simulation model, provided that they can incorporate the dynamics of the triggers in their model (e.g., adjusting the contact matrix over time).

Tables S7 and S8 give numerical values and probability distributions for the epidemiological parameters and hospitalization parameters that we use. Table S6 shows how overall age-risk group contact matrices are computed, as a function of time, based on individual contact matrices, which involve *home*, *school*, *work*, and *other* and further based on school closures, cocooning, and social distancing decisions. The specific contact matrices are then given in Tables S9-S12. After reporting additional analysis of trigger-based policies, the next section also details estimates of

age-risk populations.

Model Fitting:
Most of the parameters in our epidemiological model are based on published estimates or information from local hospitals, as detailed in the *Model Parameters* section below. However, we estimate three key parameters by fitting the model to COVID-19 hospitalization counts for the Austin-Round Rock MSA from March 13 to April 16, 2020. The data were provided directly by all area hospitals to the Austin Executive COVID-19 Task Force.

Specifically, we use the local hospitalization data to fit the following model parameters: (i) the seeding date of the epidemic (i.e., a plausible date for a case importation that sparked the ensuing epidemic; this does not rule out the possibility of earlier importations that produced only limited clusters of cases); (ii) the transmission parameter, $\beta$; and (iii) the transmission reduction during the SHWSO (March 25–May 1), denoted by $\kappa$. We use the hospitalization totals (i.e., compartment $IH$ aggregated across age and risk groups) to fit these parameters, as this is a variable for which we have data observed daily. Our results estimate the start date to be on February 15, 2020, a baseline $\beta = 0.0351$ (given in Table S7), and a transmission reduction of $\kappa = 93.65\%$ which we rounded to $\kappa = 95\%$ during the SHWSO, meaning that transmission is reduced to 5% of its baseline value.

We fit the three parameters using a least squares algorithm. We search for values of the triple that minimizes the sum of squared daily differences between the simulated, $\hat{IH}_t$, and observed, $IH_t$, daily hospitalizations between March 13 and April 16, 2020: $\sum_t (\hat{IH}_t - IH_t)^2$. For a given triple of parameter values, we use our deterministic model to simulate $\hat{IH}_t$, assuming fixed values for all other parameters, set to their mean, if they are random (see Table S7). We sequentially sweep through a range of initial seed dates, ranging from February 1 to 29 using a one-day increment; for each date we use least-squares minimization via SciPy/Python (3) to solve for $\beta$ and $\kappa$. We then select the triple that produces the smallest (least squares) discrepancy between the model estimates and the observed data.

The following are key dates, all in 2020:

- February 15: Seeding date for the first pandemic wave in Austin, assuming seeding by a single symptomatic individual age 18-49y. The initial transmission rate is $\beta$. Both the seeding date and $\beta$ are estimated from the hospitalization data.
- March 13: First reported COVID-19 hospitalization in Austin, first date of hospitalization data used to estimate the seed date, $\kappa$, and $\beta$.
- March 14: Major school districts in Austin closed school through August 17, 2020. Transmission is reduced in the model via elimination of school-based contacts from age-specific contact matrices. (Table S9-S12);
- March 24: Austin's Stay Home-Work Safe Order is enacted at midnight (4).
- March 25: The transmission rate decreases due to the SHWSO, by a factor of $\kappa$, which is estimated from the hospitalization data.
- April 16: Last date of observed hospitalizations used to estimate the seed date, $\kappa$, and $\beta$.
- May 1: The governor of Texas relaxed social distancing orders statewide (5).
- Holidays and no school events are summarized in Table S13.

**Supplementary Analysis.** We report additional results using the same format as the primary figure in the main text. For easy reference, Figure S2a repeats the results for the optimized two-level trigger for the baseline analysis; i.e., we assume 95% effective cocooning, and we toggle between 90% SD (lock-down) and 40% SD (relaxation). The daily hospitalization thresholds, and the timing of the transition, are optimized using the model of Eq. [4]. We fix the safety trigger that prevents premature relaxation at 60% of hospital capacity. Part (b) of Figure S2 repeats this optimization, but instead restricts solutions to a *constant* rather than two-level threshold. Parts (c) and (d) show the results of *stress tests*, in which we assess the performance of the optimal strategies shown in parts (a) and (b) when relaxed social distancing reduces transmission by only 20% rather than 40%. Under the two-level trigger, the point forecast remains under hospital capacity but the distributional forecasts suggest a significant probability that hospitals will be at, or just beyond, capacity at the peak in July. Under the constant trigger, even the point forecast "mildly" exceeds hospital capacity.

Figure S3 performs an analogous stress test with respect to cocooning. First, we optimize for our baseline 90%–40% SD toggle, under the assumption that cocooning is 95% effective (as in Figure S2a). Then, we evaluate the performance under the assumption that cocooning is only 80% (rather than 95%) effective. Whereas our optimal policy is robust to variation in the overall efficacy of social distancing (Figure S2), it is highly sensitive to the efficacy of cocooning. This slight reduction in cocooning is expected to lead to catastrophic surges in hospitalizations, with capacity grossly exceeded in the summer and early fall of 2020. This reinforces one of our primary conclusions, that vigilant cocooning of vulnerable populations will be critical to preventing overwhelming healthcare surges and saving lives.

To quantify the benefit of deriving optimal triggers versus sensible expert-designed strategies, we consider two reasonable *constant* thresholds for initiating and relaxing social distancing. Part (a) of Figure S4 projects pandemic waves and lock-down periods based on an arbitrary threshold that is below the optimal constant threshold shown in Figure S2c and Part (b) similarly considers an arbitrary threshold above the optimal value.

Finally, we consider a scenario in which transmission is more effectively mitigated during relaxation of social distancing. When lock-downs are lifted, the population continues to reduce transmission by 80% rather than 40%. Figure S5 projects COVID-19 under a policy that has been optimized under this more optimistic scenario. The model projects that hospitalizations will remain under capacity without requiring another lock-down before September 2021 because of the stringent mitigation even during the relaxation period.

Table S1 is the analog of Tables 1-3 from the main text, except that it reports values for projected days of lock-down, probabilities of exceeding hospital capacity, and COVID-19 mortality under the policies for Figure 1(a) and 1(b) of the main document. Tables S2 and S3 similarly report details for Figures S2a-S2b and S2c-S2d, respectively. Table S4 does so for Figures S4a-S4b, and Table S5 finally repeats these details for Figures S3 and S5.

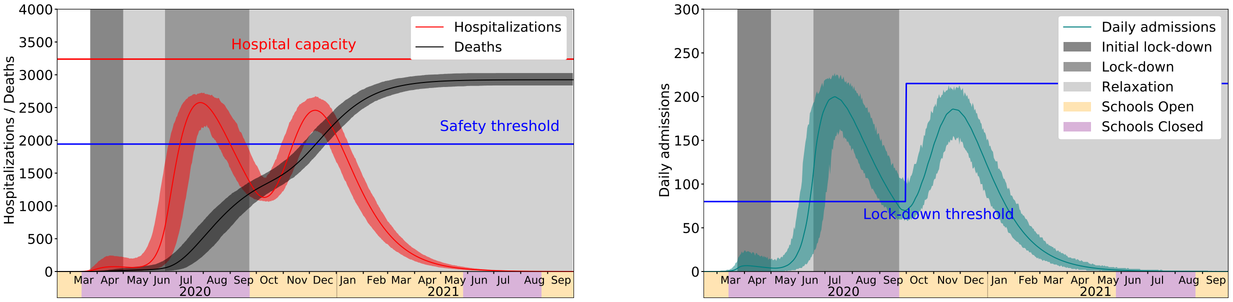


**(a)** Optimized two-level threshold

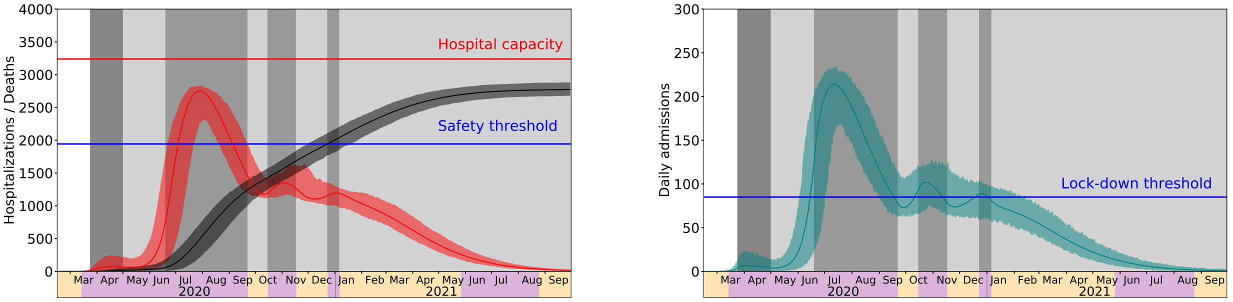


**(b)** Optimized constant threshold

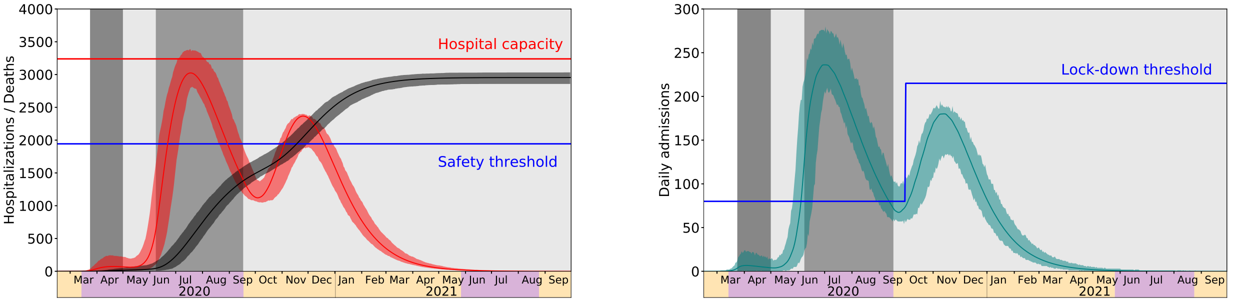


**(c)** Stress test: transmission reduced 20% instead of 40% during relaxation (two-level threshold)

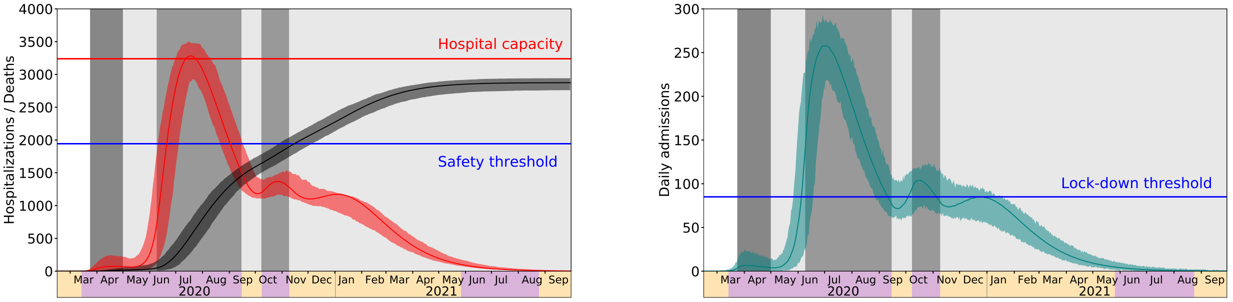


**(d)** Stress test: transmission reduced 20% instead of 40% during relaxation (constant threshold)

**Fig. S2.** Stress test of optimized strategies with respect to transmission rates following the relaxation of social distancing. The graphs project COVID-19 hospitalizations and deaths in the Austin, Texas metropolitan area through September of 2021 under optimized policies for initiating and relaxing social distancing measures, assuming 95% effective cocooning of vulnerable populations. Left-hand panels show daily hospitalizations and cumulative deaths. COVID-19 surge capacity in Austin is approximately 3240 beds (red line). Right-hand panels show daily hospital admissions. Optimized strategies use a stepped threshold: lock-downs are enacted when the seven-day rolling average in daily admissions surpasses a threshold and are relaxed when admissions decline below a threshold (indicated with blue horizontal lines), if hospitalizations are below 60% capacity. Note that the cyan curves indicate daily admissions rather than seven-day averages and thus changes (indicated by horizontal gray regions) are triggered a few days after the daily values cross a threshold. Part (a) repeats a figure presented in the main text showing the optimal two-step threshold. Part (b) instead uses a constant threshold. Parts (c) and (d) implement the policies optimized in parts (a) and (b), respectively. However, they assume that the public adheres only to 20% social distancing rather than the 40% for which the policy was designed. Hospital capacity may be exceeded if transmission rates are higher than expected during periods of relaxation. In all graphs, solid curves correspond to the point forecast and shaded regions give 90% prediction intervals based on 300 stochastic simulations.

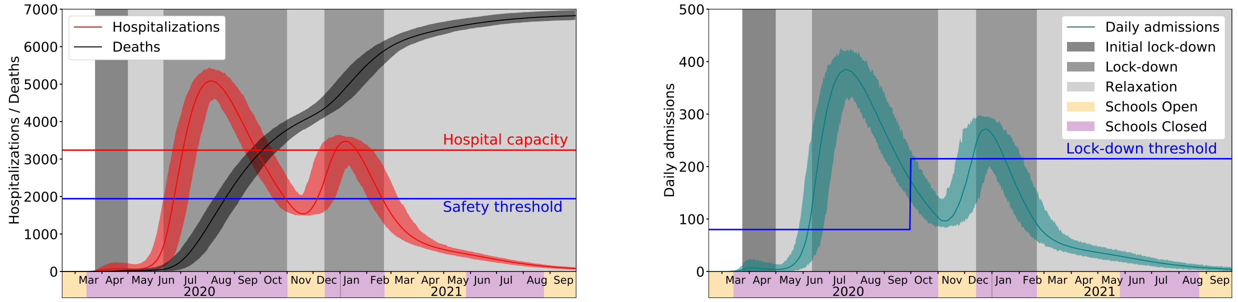


**Fig. S3.** Stress test of optimized strategies with respect to the efficacy of cocooning vulnerable populations. The graphs project COVID-19 hospitalizations and deaths in the Austin, Texas metropolitan area through September of 2021 under optimized policies for initiating and relaxing social distancing measures. The left-hand panel show daily hospitalizations and cumulative deaths. COVID-19 surge capacity in Austin is approximately 3240 beds (red line). Lock-downs can only relax if the total hospitalizations are below a safety threshold of 60% capacity (blue line). The right-hand panel show daily hospital admissions. Lock-downs are enacted when daily admissions (seven-day rolling average) surpass the indicated thresholds and are relaxed when admissions decline below the thresholds, if hospitalizations are below 60% capacity. To make these graphs, we first derived the optimal thresholds for the baseline scenario which toggles between 90% (lock-down) and 40% (relaxation) transmission reduction, while assuming that cocooning remains 95% effective. Then, we evaluate performance of the triggering policy under the assumption that cocooning is only 80% effective in reducing infection risk in vulnerable populations. Imperfect cocooning is projected to result in catastrophic health care surges and more than double the mortality. In both graphs, solid curves correspond to the point forecast and shaded regions give 90% prediction intervals based on 300 stochastic simulations.

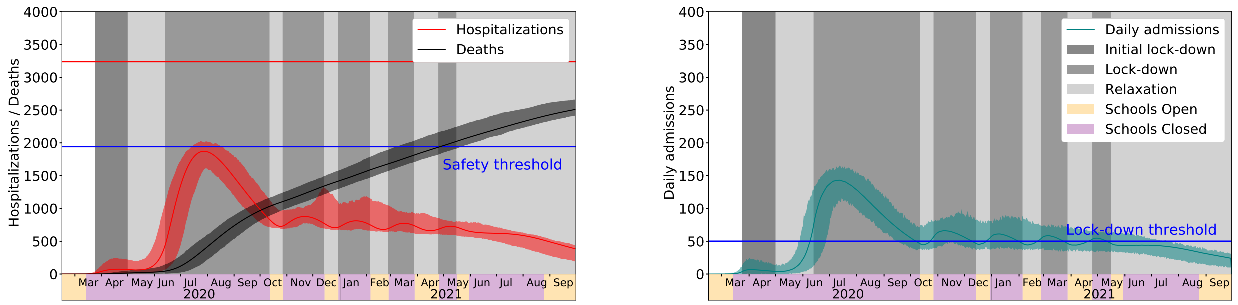


**(a)** Arbitrary lock-down threshold of 50 hospital admissions per day

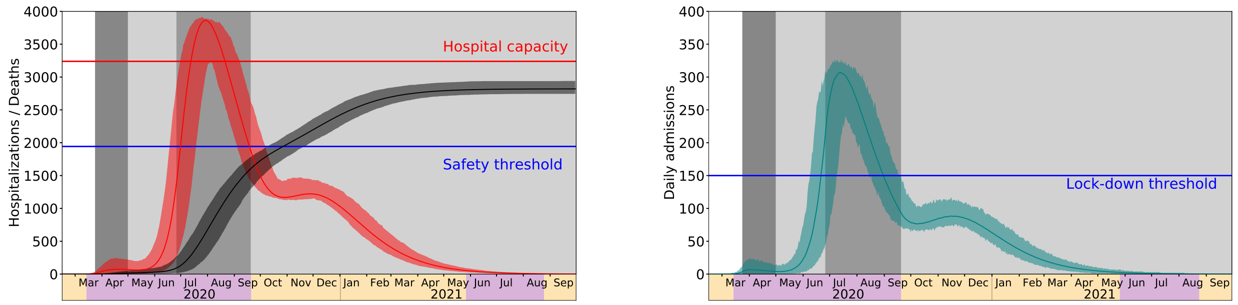


**(b)** Arbitrary lock-down threshold of 150 hospital admissions per day

**Fig. S4.** Non-optimized thresholds for triggering COVID-19 lock-downs. The graphs project COVID-19 hospitalizations and deaths in the Austin, Texas metropolitan area through September of 2021 under arbitrarily chosen triggers for initiating and relaxing social distancing measures, assuming 95% effective cocooning of vulnerable populations. Left-hand panels show daily hospitalizations and cumulative deaths. COVID-19 surge capacity in Austin is approximately 3240 beds (red line). Lock-downs can only relax if the total hospitalizations are below a safety threshold of 60% capacity (blue line). Right-hand panels show daily hospital admissions. Lock-downs are enacted when daily admissions (seven-day rolling average) surpass the indicated thresholds and are relaxed when admissions decline below the thresholds, if hospitalizations are below 60% capacity. Parts a and b assume arbitrary triggers that are below and above the optimal constant threshold from Figure S2c, respectively. In all graphs, solid curves correspond to the point forecast and shaded regions give 90% prediction intervals based on 300 stochastic simulations.

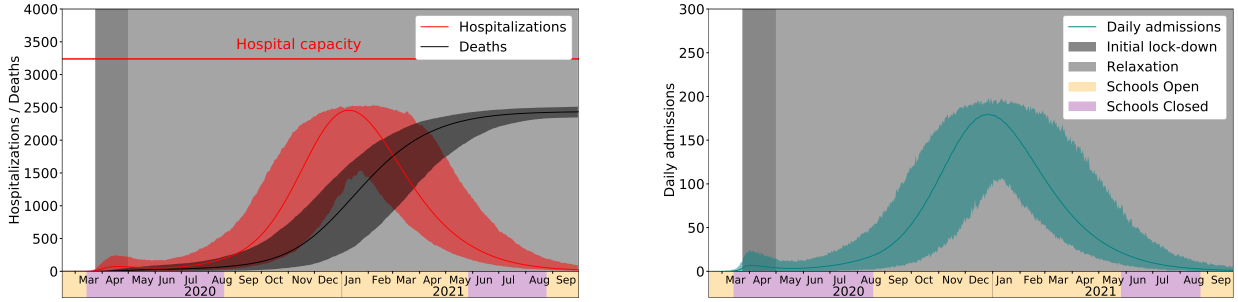


**Fig. S5.** Projections assuming an 80% rather than 40% reduction in transmission during relaxation periods. The graphs project COVID-19 hospitalizations and deaths in the Austin, Texas metropolitan area through September of 2021 under optimized policies for initiating and relaxing social distancing measures. The left-hand panel shows daily hospitalizations and cumulative deaths. If Austin maintains 80% reduction in transmission throughout the relaxation period, then hospitalizations will remain under the local COVID-19 surge capacity of approximately 3240 beds (red line) without requiring a lock-down prior to September 2021. The right-hand panel shows daily hospital admissions. In both graphs, solid curves correspond to the point forecast and shaded regions give 90% prediction intervals based on 300 stochastic simulations.

| Scenario | | Indefinite lock-down | Indefinite relaxation |
|---|---|---|---|
| Days of lock-down | Mean | 555 | 38 |
| | Median | 555 | 38 |
| | 5%-95% PI | (555-555) | (38-38) |
| Cumulative deaths by Sep 30, 2021 | Mean | 81 | 2957 |
| | Median | 65 | 2956 |
| | 5%-95% PI | (10-202) | (2868-3040) |
| Risk Group | Age Group | % deaths | |
| Low risk | 0-4y | 0.00 | 0.03 |
| | 5-17y | 0.00 | 0.20 |
| | 18-49y | 6.17 | 9.13 |
| | 50-64y | 11.11 | 18.67 |
| | 65y+ | 4.94 | 4.43 |
| High risk | 0-4y | 0.00 | 0.00 |
| | 5-17y | 0.00 | 0.10 |
| | 18-49y | 7.41 | 5.95 |
| | 50-64y | 30.86 | 26.24 |
| | 65y+ | 39.51 | 35.24 |
| Probability of exceeding % of hospital capacity | 60% | 0.000 | 1.000 |
| | 70% | 0.000 | 1.000 |
| | 80% | 0.000 | 1.000 |
| | 90% | 0.000 | 1.000 |
| | 100% | 0.000 | 1.000 |

**Table S1. Projected days of lock-down, probabilities of exceeding hospital capacity and COVID-19 mortality under the indefinite lock-down and indefinite relaxation. Companion of Figure 1(a) and 1(b) of the main document.**

| Scenario | | Stepped threshold | Constant threshold |
|---|---|---|---|
| Days of lock-down | Mean | 135 | 179 |
| | Median | 135 | 179 |
| | 5%-95% PI | (126-141) | (170-189) |
| Cumulative deaths by Sep 30, 2021 | Mean | 2929 | 2781 |
| | Median | 2646 | 2778 |
| | 5%-95% PI | (2837-3026) | (2679-2883) |
| Risk Group | Age Group | % deaths | |
| Low risk | 0-4y | 0.03 | 0.04 |
| | 5-17y | 0.20 | 0.22 |
| | 18-49y | 9.01 | 9.17 |
| | 50-64y | 18.23 | 18.01 |
| | 65y+ | 4.43 | 4.42 |
| High risk | 0-4y | 0.00 | 0.00 |
| | 5-17y | 0.10 | 0.07 |
| | 18-49y | 6.04 | 6.08 |
| | 50-64y | 26.86 | 26.68 |
| | 65y+ | 35.06 | 35.31 |
| Probability of exceeding % of hospital capacity | 60% | 1.000 | 1.000 |
| | 70% | 0.997 | 1.000 |
| | 80% | 0.803 | 0.917 |
| | 90% | 0.020 | 0.030 |
| | 100% | 0.000 | 0.000 |

**Table S2. Projected days of lock-down, probabilities of exceeding hospital capacity and COVID-19 mortality under the optimized stepped threshold and the optimized constant threshold. These correspond to Figures S2a and S2b in which relaxed social distancing corresponds to a 40% reduction in transmission.**

| Scenario | | Stepped threshold | Constant threshold |
|---|---|---|---|
| Days of lock-down | Mean | 139 | 173 |
| | Median | 139 | 173 |
| | 5%-95% PI | (136-144) | (165-185) |
| Cumulative deaths by Sep 30, 2021 | Mean | 2950 | 2852 |
| | Median | 2948 | 2855 |
| | 5%-95% PI | (2857-3033) | (2761-2942) |
| Risk Group | Age Group | % deaths | |
| Low risk | 0-4y | 0.03 | 0.04 |
| | 5-17y | 0.20 | 0.21 |
| | 18-49y | 9.05 | 9.15 |
| | 50-64y | 18.27 | 18.16 |
| | 65y+ | 4.44 | 4.45 |
| High risk | 0-4y | 0.00 | 0.00 |
| | 5-17y | 0.10 | 0.11 |
| | 18-49y | 6.00 | 5.96 |
| | 50-64y | 26.68 | 26.61 |
| | 65y+ | 35.22 | 35.31 |
| Probability of exceeding % of hospital capacity | 70% | 1.00 | 1.000 |
| | 80% | 0.997 | 0.997 |
| | 90% | 0.983 | 0.997 |
| | 100% | 0.437 | 0.797 |
| | 110% | 0.003 | 0.053 |

**Table S3. Projected days of lock-down, probabilities of exceeding hospital capacity and COVID-19 mortality under stress tests for the stepped threshold and the constant threshold. These correspond to Figures S2c and S2d. Policies are trained assuming that relaxed social distancing corresponds to a 40% reduction in transmission, but then tested when relaxed social distancing instead corresponds to a 20% reduction in transmission.**

| Scenario | | Threshold of 50 | Threshold of 150 |
|---|---|---|---|
| Days of lock-down | Mean | 291 | 123 |
| | Median | 292 | 124 |
| | 5%-95% PI | (270-308) | (121-125) |
| Cumulative deaths by Sep 30, 2021 | Mean | 2526 | 2839 |
| | Median | 2520 | 2837 |
| | 5%-95% PI | (2416-2660) | (2744-2940) |
| Risk Group | Age Group | % deaths | |
| Low risk | 0-4y | 0.04 | 0.04 |
| | 5-17y | 0.20 | 0.21 |
| | 18-49y | 9.34 | 9.16 |
| | 50-64y | 17.93 | 18.07 |
| | 65y+ | 4.43 | 4.44 |
| High risk | 0-4y | 0.00 | 0.00 |
| | 5-17y | 0.08 | 0.11 |
| | 18-49y | 6.02 | 6.02 |
| | 50-64y | 26.72 | 26.70 |
| | 65y+ | 35.23 | 35.26 |
| Probability of exceeding % of hospital capacity | 60% | 0.293 | 1.000 |
| | 70% | 0.000 | 1.000 |
| | 80% | 0.000 | 1.000 |
| | 90% | 0.000 | 1.000 |
| | 100% | 0.000 | 1.000 |

**Table S4. Projected days of lock-down, probabilities of exceeding hospital capacity and COVID-19 mortality under non-optimized constant thresholds. These correspond to Figures S4a and S4b. Relaxed social distancing corresponds to a 40% reduction in transmission.**

| Scenario | | 80% Cocooning | 80% Relaxed |
|---|---|---|---|
| Days of lock-down | Mean | 249 | 38 |
| | Median | 249 | 38 |
| | 5%-95% PI | (243-254) | (38-38) |
| Cumulative deaths by Sep 30, 2021 | Mean | 6839 | 2426 |
| | Median | 6830 | 2427 |
| | 5%-95% PI | (6715-6977) | (2346-2511) |
| Risk Group | Age Group | % deaths | |
| Low risk | 0-4y | 0.04 | 0.04 |
| | 5-17y | 0.20 | 0.21 |
| | 18-49y | 9.34 | 8.90 |
| | 50-64y | 17.93 | 16.61 |
| | 65y+ | 4.43 | 4.45 |
| High risk | 0-4y | 0.00 | 0.00 |
| | 5-17y | 0.08 | 0.12 |
| | 18-49y | 6.02 | 6.43 |
| | 50-64y | 26.72 | 28.03 |
| | 65y+ | 35.23 | 35.20 |
| Probability of exceeding % of hospital capacity | 60% | 1.000 | 0.997 |
| | 70% | 1.000 | 0.993 |
| | 80% | 1.000 | 0.080 |
| | 90% | 1.000 | 0.000 |
| | 100% | 1.000 | 0.000 |

**Table S5. Projected days of lock-down, probabilities of exceeding hospital capacity and COVID-19 mortality under two scenarios. The first is a stress test, which optimizes assuming cocooning is 95% effective, but tests when it is instead 80% effective; this corresponds to Figure S3. The second policy corresponds to relaxed social distancing corresponding to 80% (rather than our nominal 40%) reduction in transmission under cocooning, which is 95% effective. In this case we do not need to enforce a lock-down after May 1, 2020 to prevent hospitalizations from exceeding capacity. The right-most column corresponds to Figure S5.**

**Model Parameters.**

Age-stratified proportion of population at high-risk for COVID-19 complications:
We estimate age-specific proportions of the population at high risk of complications from COVID-19 based on data for Austin, TX and Round-Rock, TX from the CDC's 500 cities project (Figure S6) (6). We assume that high risk conditions for COVID-19 are the same as those specified for influenza by the CDC (7). The CDC's 500 cities project provides city-specific estimates of prevalence for several of these conditions among adults (8). The estimates were obtained from the 2015-2016 Behavioral Risk Factor Surveillance System (BRFSS) data using a small-area estimation methodology called multi-level regression and poststratification (9, 10). It links geocoded health surveys to high spatial resolution population demographic and socioeconomic data (10).

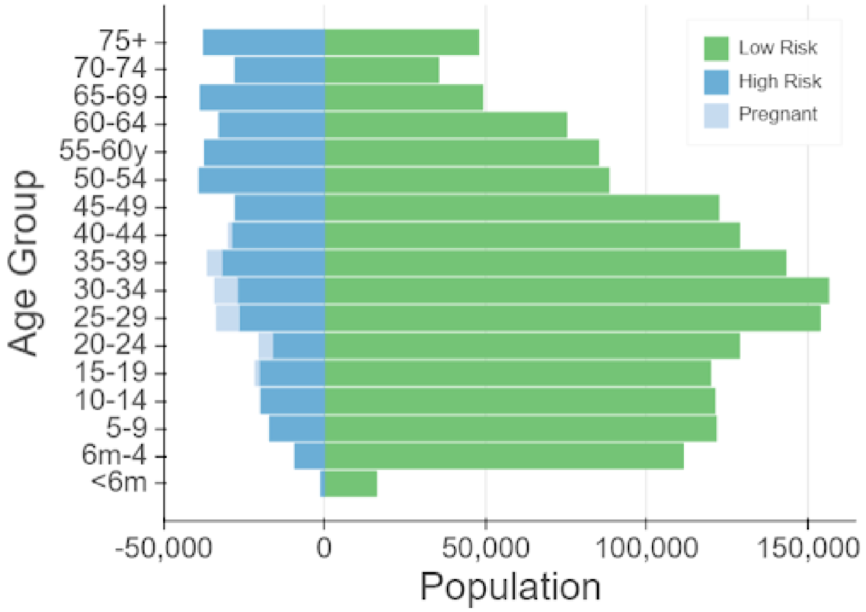


**Fig. S6. Demographic and risk composition of the Austin-Round Rock MSA.** Bars indicate age-specific population sizes, separated by low risk, high risk, and pregnant women. High risk is defined as individuals with cancer, chronic kidney disease, chronic obstructive pulmonary disease (COPD), heart disease, stroke, asthma, diabetes, HIV/AIDS, and morbid obesity.

High-risk proportions for adults:
To estimate the proportion of adults at high risk for complications, we use the CDC's 500 cities data mentioned above, as well as data on the prevalence of HIV/AIDS, obesity and pregnancy among adults (Table S14).

The CDC 500 cities dataset includes the prevalence of each condition on its own, rather than the prevalence of multiple conditions (e.g., dyads or triads). Thus, we use separate co-morbidity estimates to determine overlap. The following reference about chronic conditions (11) gives US estimates for the proportion of the adult population with 0, 1 or 2+ chronic conditions, per age group. Using this and the 500 cities data we can estimate the proportion of the population $p_{HR}$ in each age group in each city with at least one chronic condition listed in the CDC 500 cities data (Table S14) putting them at high-risk for flu complications.

HIV:
We use the data from Table 20a in CDC HIV surveillance report (12) to estimate the population in each risk group living with HIV in the US (last column, 2015 data). Assuming independence between HIV and other chronic conditions, we increase the proportion of the population at high-risk for influenza to account for individuals with HIV but no other underlying conditions.

Morbid obesity:
A BMI over 40kg/m$^2$ indicates morbid obesity, and is considered high risk for influenza. The 500 Cities Project reports the prevalence of obese people in each city with BMI over 30kg/m2 (not necessarily morbid obesity). We use the data from Table 1 in (13) to estimate the proportion of people with BMI $> 30$ that actually have BMI$> 40$ (across the US); we then apply this to the 500 Cities obesity data to estimate the proportion of people who are morbidly obese in each city. Table 1 of (13) suggests that 51.2% of morbidly obese adults have at least one other high risk

chronic condition, and update our high-risk population estimates accordingly to account for overlap.

Pregnancy:

We separately estimate the number of pregnant women in each age group and each city, following the methodology in a CDC reproductive health report (14). We assume independence between any of the high-risk factors and pregnancy, and further assume that half the population are women; also, see (15).

Estimating high-risk proportions for children:

Since the 500 Cities Project only reports data for adults 18 years and older, we take a different approach to estimating the proportion of children at high risk for severe influenza. The two most prevalent risk factors for children are asthma and obesity; we also account for childhood diabetes, HIV and cancer. From (16), we obtain national estimates of chronic conditions in children. For asthma, we assume that variation among cities will be similar for children and adults. Thus, we use the relative prevalence of asthma in adults to scale our estimates for children in each city. The prevalence of HIV and cancer in children are taken from CDC HIV surveillance report (12) and cancer research report (17), respectively.

We first estimate the proportion of children having either asthma, diabetes, cancer or HIV (assuming no overlap in these conditions). We estimate city-level morbid obesity in children using the estimated morbid obesity in adults multiplied by a national constant ratio for each age group estimated from (18), this ratio represents the prevalence in morbid obesity in children given the one observed in adults. From (19), we estimate that 25% of morbidly obese children have another high-risk condition and adjust our final estimates accordingly.

Resulting estimates: We compare our estimates for the Austin-Round Rock Metropolitan Area to published national-level estimates (20) of the proportion of each age group with underlying high risk conditions (Table S14). The biggest difference is observed in older adults, with Austin having a lower proportion at risk for complications for COVID-19 than the national average; for 25-39 year olds the high risk proportion is slightly higher than the national average.

| Variable | Settings |
|---|---|
| Initial day of simulation | 2/15/2020 |
| Initial infection number in locations | 1 symptomatic case in 18-49 year age group |
| Social distancing reduction in contacts | As marked in the corresponding table and figure. Note that social distancing is determined from the triggers. |
| Age-specific and day-specific contact rates | Home, work, other and school matrices provided in Tables S9-S12<br>As marked in corresponding table and figures with social level distance denoted as SD and cocooning level denoted as CO:<br>If no measure is in place (other than social distance):<br>Weekday = (1-SD) (home + school +work + other)<br>Weekend = (1-SD) (home + other)<br>If only schools are closed:<br>Weekday = (1-SD) (home + work + other)<br>Weekend = (1-SD) (home + other)<br>If only cocooning is on:<br>Low risk and age groups 1-4<br>Weekday = (1-SD) (home + school + work + other)<br>Weekend = (1-SD) (home + other)<br>High risk and age group 5<br>Weekday = (1-CO) (home + school + work + other)<br>Weekend = (1-CO) (home + other)<br>If schools are closed and cocooning is on:<br>Low risk and age groups 1-4<br>Weekday = (1-SD) (home + work + other)<br>Weekend = (1-SD) (home + other)<br>High risk and age group 5<br>Weekday = (1-CO) (home + work + other)<br>Weekend = (1-CO) (home + other) |

**Table S6. Initial conditions, school closures and social distancing policies**

| Parameters | Best guess values | Source |
|---|---|---|
| $\beta$: transmission rate | 0.0351 | Fitted [*] to daily COVID-19 hospitalizations in Austin-Round Rock MSA |
| $R_0$: basic reproductive number | 10.3[1] | Calculated as a function of other epidemiological parameters |
| $\gamma_A$: recovery rate from compartment $IA$ | Equal to $\gamma_Y$ | |
| $\gamma_Y$: recovery rate from symptomatic compartment $IY$ | $\frac{1}{\gamma} \sim$ Triangular (21.2, 22.6, 24.4) | (21) |
| $\tau$ : symptomatic proportion (%) | 82.1 | (22) |
| $\sigma$ : exposed rate | $\frac{1}{\sigma} \sim$ Triangular (5.6,7.0,8.2) | (23) |
| $P$: proportion of pre-symptomatic transmission (%) | 12.6 | (24) |
| $\omega_E$: infectiousness of individuals in compartment $E$, relative to $IY$ | $\omega_E = \frac{(\frac{YHR}{\eta} + \frac{1-YHR}{\gamma_Y})\sigma P}{1-P}$ | |
| $\omega_A$: infectiousness of individuals in compartment $IA$, relative to $IY$ | 0.4653 | Set to mean of $\omega_E$ |
| $IFR$: infected fatality ratio, age specific (%) | Low risk / High risk<br>0.0009 / 0.0092<br>0.00222 / 0.0218<br>0.0339 / 0.3388<br>0.2520 / 2.5197<br>0.6440 / 6.4402 | Age adjusted from (21) |
| $YFR$: symptomatic fatality ratio, age specific (%) | Low risk / High risk<br>0.0011165 / 0.0112<br>0.0027 / 0.0265<br>0.0412 / 0.4126<br>0.3069 / 3.069<br>0.7844 / 7.8443 | $YFR = \frac{IFR}{1-\tau}$ |
| $h$: high-risk proportion, age specific (%) | 8.2825<br>14.1121<br>16.5298<br>32.9912<br>47.0568 | Estimated using 2015-2016 Behavioral Risk Factor Surveillance System (BRFSS) data with multilevel regression and poststratification using CDC's list of conditions that may increase the risk of serious complications from influenza (9, 10) |

**Table S7. Model parameters**

[1] The basic reproduction number ($R_0$) is derived from the fixed and fit parameters listed in Tables S7 and S8 using a next-generation matrix approach (25). Prior to March 14, 2020 (i.e., prior to school closures and the subsequent stay-home order) we calculate $R_0 = 10.3$, and under a 95% reduction in transmission and schools closed the estimated $R_0$ drops to 0.44. The baseline (unmitigated) estimate is higher than most recent published estimates, which likely stems from our assumption regarding the duration of the infectious period which we based on early estimates out of China (21). A more current version of the model, which has been updated to include a shorter period of acute infectiousness beginning a couple days before symptom onset, suggests $R_0 = 5.1$ prior to March 14th.

| Parameters | Value | | Source |
|---|---|---|---|
| $\gamma_H$ : recovery rate in compartment $IH$ | 1/14 | | 14 day-average from admission to discharge (UT Austin Dell Med) |
| | Low risk | High risk | |
| $YHR$: symptomatic case hospitalization rate (%) | 0.0279<br>0.0215<br>1.3215<br>2.8563<br>3.3873 | 0.2791<br>0.2146<br>13.2514<br>28.5634<br>33.873 | Age adjusted from (21) |
| $\pi$ : rate symptomatic individuals go to hospital, age-specific | $\pi = \frac{\gamma_Y * YHR}{\eta + (\gamma_Y - \eta) YHR}$ | | |
| $\eta$ : rate from symptom onset to hospitalized | 0.1695 | | 5.9 day average from symptom onset to hospital admission (26) |
| $\mu$ : rate from hospitalized to death | 1/14 | | 14 day-average from admission to death (UT Austin Dell Med) |
| $HFR$: hospitalized fatality ratio, age specific (%) | 4.000<br>12.365<br>3.122<br>10.745<br>23.158 | | $HFR = \frac{IFR}{YHR(1-\tau)}$ |
| $\nu$ : death rate on hospitalized individuals, age specific | 0.0390<br>0.1208<br>0.0304<br>0.1049<br>0.2269 | | $\nu = \frac{\gamma_H * HFR}{\mu + (\gamma_H - \mu) HFR}$ |
| $B$: Healthcare capacity | Hospital bed: 4049 (assume 80% of these are available for COVID-19) | | Estimates provided by each of the region's hospital systems and aggregated by regional public health leaders |

**Table S8. Hospitalization parameters**

| | 0-4y | 5-17y | 18-49y | 50-64y | 65y+ |
|---|---|---|---|---|---|
| 0-4y | 0.5 | 0.9 | 2.0 | 0.1 | 0.0 |
| 5-17y | 0.2 | 1.7 | 1.9 | 0.2 | 0.0 |
| 18-49y | 0.2 | 0.9 | 1.7 | 0.2 | 0.0 |
| 50-64y | 0.2 | 0.7 | 1.2 | 1.0 | 0.1 |
| 65y+ | 0.1 | 0.7 | 1.0 | 0.3 | 0.6 |

**Table S9. Home contact matrix. Daily number contacts by age group at home.**

| | 0-4y | 5-17y | 18-49y | 50-64y | 65y+ |
|---|---|---|---|---|---|
| 0-4y | 1.0 | 0.5 | 0.4 | 0.1 | 0.0 |
| 5-17y | 0.2 | 3.7 | 0.9 | 0.1 | 0.0 |
| 18-49y | 0.0 | 0.7 | 0.8 | 0.0 | 0.0 |
| 50-64y | 0.1 | 0.8 | 0.5 | 0.1 | 0.0 |
| 65y+ | 0.0 | 0.0 | 0.1 | 0.0 | 0.0 |

**Table S10. School contact matrix. Daily number contacts by age group at school.**

| | 0-4y | 5-17y | 18-49y | 50-64y | 65y+ |
|---|---|---|---|---|---|
| 0-4y | 0.0 | 0.0 | 0.0 | 0.0 | 0.0 |
| 5-17y | 0.0 | 0.1 | 0.4 | 0.0 | 0.0 |
| 18-49y | 0.0 | 0.2 | 4.5 | 0.8 | 0.0 |
| 50-64y | 0.0 | 0.1 | 2.8 | 0.9 | 0.0 |
| 65y+ | 0.0 | 0.0 | 0.1 | 0.0 | 0.0 |

**Table S11. Work contact matrix. Daily number contacts by age group at work.**

| | 0-4y | 5-17y | 18-49y | 50-64y | 65y+ |
|---|---|---|---|---|---|
| 0-4y | 0.7 | 0.7 | 1.8 | 0.6 | 0.3 |
| 5-17y | 0.2 | 2.6 | 2.1 | 0.4 | 0.2 |
| 18-49y | 0.1 | 0.7 | 3.3 | 0.6 | 0.2 |
| 50-64y | 0.1 | 0.3 | 2.2 | 1.1 | 0.4 |
| 65y+ | 0.0 | 0.2 | 1.3 | 0.8 | 0.6 |

**Table S12. Others contact matrix. Daily number contacts by age group at other locations.**

| Date | Event | Date | Event | Date | Event |
|---|---|---|---|---|---|
| 01-01-2020 | Holiday | 07-16-2020 | No School | 05-27-2021 | No School |
| 01-02-2020 | No School | 07-17-2020 | No School | 05-28-2021 | No School |
| 01-03-2020 | No School | 07-20-2020 | No School | 05-31-2021 | Holiday |
| 01-04-2020 | No School | 07-21-2020 | No School | 06-01-2021 | No School |
| 01-05-2020 | No School | 07-22-2020 | No School | 06-02-2021 | No School |
| 01-06-2020 | No School | 07-23-2020 | No School | 06-03-2021 | No School |
| 01-20-2020 | Holiday | 07-24-2020 | No School | 06-04-2021 | No School |
| 02-17-2020 | Holiday | 07-27-2020 | No School | 06-07-2021 | No School |
| 03-16-2020 | Holiday | 07-28-2020 | No School | 06-08-2021 | No School |
| 03-17-2020 | Holiday | 07-29-2020 | No School | 06-09-2021 | No School |
| 03-18-2020 | Holiday | 07-30-2020 | No School | 06-10-2021 | No School |
| 03-19-2020 | Holiday | 07-31-2020 | No School | 06-11-2021 | No School |
| 03-20-2020 | Holiday | 08-03-2020 | No School | 06-14-2021 | No School |
| 04-10-2020 | Holiday | 08-04-2020 | No School | 06-15-2021 | No School |
| 05-25-2020 | Holiday | 08-05-2020 | No School | 06-16-2021 | No School |
| 05-28-2020 | No School | 08-06-2020 | No School | 06-17-2021 | No School |
| 05-29-2020 | No School | 08-07-2020 | No School | 06-18-2021 | No School |
| 06-01-2020 | No School | 08-10-2020 | No School | 06-21-2021 | No School |
| 06-02-2020 | No School | 08-11-2020 | No School | 06-22-2021 | No School |
| 06-03-2020 | No School | 08-12-2020 | No School | 06-23-2021 | No School |
| 06-04-2020 | No School | 08-13-2020 | No School | 06-24-2021 | No School |
| 06-05-2020 | No School | 08-14-2020 | No School | 06-25-2021 | No School |
| 06-08-2020 | No School | 08-17-2020 | No School | 06-28-2021 | No School |
| 06-09-2020 | No School | 09-07-2020 | Holiday | 06-29-2021 | No School |
| 06-10-2020 | No School | 10-12-2020 | Holiday | 06-30-2021 | No School |
| 06-11-2020 | No School | 11-23-2020 | Holiday | 07-01-2021 | No School |
| 06-12-2020 | No School | 11-24-2020 | Holiday | 07-02-2021 | No School |
| 06-15-2020 | No School | 11-25-2020 | Holiday | 07-05-2021 | No School |
| 06-16-2020 | No School | 11-26-2020 | Holiday | 07-06-2021 | No School |
| 06-17-2020 | No School | 11-27-2020 | Holiday | 07-07-2021 | No School |
| 06-18-2020 | No School | 12-21-2020 | No School | 07-08-2021 | No School |
| 06-19-2020 | No School | 12-22-2020 | No School | 07-09-2021 | No School |
| 06-22-2020 | No School | 12-23-2020 | No School | 07-12-2021 | No School |
| 06-23-2020 | No School | 12-24-2020 | Holiday | 07-13-2021 | No School |
| 06-24-2020 | No School | 12-25-2020 | Holiday | 07-14-2021 | No School |
| 06-25-2020 | No School | 12-28-2020 | No School | 07-15-2021 | No School |
| 06-26-2020 | No School | 12-29-2020 | No School | 07-16-2021 | No School |
| 06-29-2020 | No School | 12-30-2020 | No School | 07-19-2021 | No School |
| 06-30-2020 | No School | 12-31-2020 | Holiday | 07-20-2021 | No School |
| 07-01-2020 | No School | 01-01-2021 | Holiday | 07-21-2021 | No School |
| 07-02-2020 | No School | 01-04-2021 | No School | 07-22-2021 | No School |
| 07-03-2020 | No School | 01-05-2021 | No School | 07-23-2021 | No School |
| 07-06-2020 | No School | 01-18-2021 | Holiday | 07-26-2021 | No School |
| 07-07-2020 | No School | 02-15-2021 | Holiday | 07-27-2021 | No School |
| 07-08-2020 | No School | 03-15-2021 | Holiday | 07-28-2021 | No School |
| 07-09-2020 | No School | 03-16-2021 | Holiday | 07-29-2021 | No School |
| 07-10-2020 | No School | 03-17-2021 | Holiday | 07-30-2021 | No School |
| 07-13-2020 | No School | 03-18-2021 | Holiday | 07-31-2021 | No School |
| 07-14-2020 | No School | 03-19-2021 | Holiday | | |
| 07-15-2020 | No School | 04-02-2021 | Holiday | | |

**Table S13. Event calendar of holidays and dates with no school**

| Condition | Data source |
|---|---|
| Cancer (except skin) | CDC 500 cities (7) |
| Chronic kidney disease | CDC 500 cities (7) |
| COPD | CDC 500 cities (7) |
| Coronary heart disease | CDC 500 cities (7) |
| Stroke | CDC 500 cities (7) |
| Asthma | CDC 500 cities (7) |
| Diabetes | CDC 500 cities (7) |
| HIV/AIDS | CDC HIV Surveillance Report (12) |
| Obesity | CDC 500 cities and (13, 19) |
| Pregnancy | National Vital Statistics Reports (27) and abortion data (28) |

**Table S14. High-risk conditions for influenza and data sources for prevalence estimation**

| Age Group | National estimates (18) | Austin (excluding pregnancy) | Pregnant women (proportion of age group) |
|---|---|---|---|
| 0-0.5y | n/a | 6.8 | - |
| 0.5-4y | 6.8 | 7.4 | - |
| 5-9 y | 11.7 | 11.6 | - |
| 10-14y | 11.7 | 13.0 | - |
| 15-19y | 11.8 | 13.3 | 1.7 |
| 20-24y | 12.4 | 10.3 | 5.1 |
| 25-34y | 15.7 | 13.5 | 7.8 |
| 35-39y | 15.7 | 17.0 | 5.1 |
| 40-44y | 15.7 | 17.4 | 1.2 |
| 45-49y | 15.7 | 17.7 | - |
| 50-54y | 30.6 | 29.6 | - |
| 55-60y | 30.6 | 29.5 | - |
| 60-64y | 30.6 | 29.3 | - |
| 65-69y | 47.0 | 42.2 | - |
| 70-74y | 47.0 | 42.2 | - |
| 75y+ | 47.0 | 42.2 | - |

**Table S15. Comparison between published national estimates and Austin-Round Rock MSA estimates of the percent of the population at high-risk of influenza/COVID-19 complications.**

## References


1. Halfin S, Whitt W (1981) Heavy-traffic limits for queues with many exponential servers. *Operations Research* 29(3):567–588.
2. Wang X, et al. (2020) Impact of social distancing measures on COVID-19 healthcare demand in Central Texas. *medRxiv*.
3. SciPy.org (2019) minimize(method='trust-constr').
4. The Mayor of the City of Austin (2020) STAY HOME – WORK SAFE ORDER NO. 20200413-009, The City of Austin.
5. The Governor of Texas (2020) Executive Order GA18: Relating to the expanded reopening of services as part of the safe, strategic plan to open Texas in response to the COVID-19 disaster.
6. Centers for Disease Control and Prevention (2018) People at high risk for flu complications.
7. Centers for Disease Control and Prevention (2019) 500 cities: Local data for better health.
8. Centers for Disease Control and Prevention (2016) Health outcomes.
9. Centers for Disease Control and Prevention (2019) Behavioral risk factor surveillance system.
10. Zhang X, et al. (2014) Multilevel regression and poststratification for small-area estimation of population health outcomes: a case study of chronic obstructive pulmonary disease prevalence using the behavioral risk factor surveillance system. *American Journal of Epidemiology* 179(8):1025–1033.
11. Fox S, Duggan M (2013) Part one: Who lives with chronic conditions.
12. Centers for Disease Control and Prevention (2020) HIV Surveillance Reports.
13. Sturm R, Hattori A (2013) Morbid obesity rates continue to rise rapidly in the United States. *International Journal of Obesity* 37(6):889–891.
14. CDC Division of Reproductive Health (2013) Estimating the number of pregnant women in a geographic area.
15. Singh B, Meyers LA (2017) Estimation of single-year-of-age counts of live births, fetal losses, abortions, and pregnant women for counties of Texas. *BMC Research Notes* 10(1).
16. Miller G, Coffield E, Leroy Z, Wallin R (2016) Prevalence and costs of five chronic conditions in children. *The Journal of School Nursing* 32(5):357–364.
17. American Cancer Society (2020) Cancer Facts & Figures 2014.
18. Hales CM, Fryar CD, Carroll MD, Freedman DS, Ogden CL (2018) Trends in obesity and severe obesity prevalence in US youth and adults by sex and age, 2007-2008 to 2015-2016. *JAMA* 319(16):1723–1725.
19. Morgan O, et al. (2010) Morbid obesity as a risk factor for hospitalization and death due to 2009 pandemic influenza A(H1N1) disease. *PloS One* 5(3):9694.
20. Zimmerman R, Lauderdale D, Tan S, Wagener D (2010) Prevalence of high-risk indications for influenza vaccine varies by age, race, and income. *Vaccine* 28(39):6470–6477.
21. Verity R, et al. (2020) Estimates of the severity of coronavirus disease 2019: a model-based analysis. *The Lancet Infectious Diseases*.
22. Mizumoto K, Kagaya K, Zarebski A, Chowell G (2020) Estimating the asymptomatic proportion of 2019 novel coronavirus onboard the Princess Cruises Ship, 2020. *Eurosurveillance* 25(10).
23. Lauer S, et al. (2020) The incubation period of coronavirus disease 2019 (COVID-19) from publicly reported confirmed cases: Estimation and application. *Annals of Internal Medicine* 172(9):577–582.
24. Du Z, et al. (2020) Serial interval of COVID-19 among publicly reported confirmed cases. *Emerging Infectious Diseases* 26(6).
25. Diekmann O, Heesterbeek JAP RM (2009) The construction of next-generation matrices for compartmental epidemic models. *Journal of The Royal Society Interface* 7(47):873–885.
26. Tindale L, et al. (2020) Transmission interval estimates suggest pre-symptomatic spread of covid-19. *Preprint*.
27. Martin JA, Hamilton BE, Osterman MJK, Driscoll AK, Drake P (2017) Births: Final data for 2017. *National Vital Statistics Reports* 67(8).
28. Jatlaoui TC, et al. (2018) Abortion Surveillance—United States, 2014. *MMWR Surveillance Summaries* 66(25):1.
